# Integrating Frequency-Spatial Features for Energy-Efficient OPGW Target Recognition in UAV-Assisted Mobile Monitoring

**DOI:** 10.3390/s26020506

**Published:** 2026-01-12

**Authors:** Lin Huang, Xubin Ren, Daiming Qu, Lanhua Li, Jing Xu

**Affiliations:** 1 School of Electronic Information and Communications, Huazhong University of Science and Technology, Wuhan 430074, China; d201980615@hust.edu.cn (L.H.); qudaiming@hust.edu.cn (D.Q.); 2 Wuhan Maritime Communication Research Institute, Wuhan 430079, China; 3 School of Intelligent Systems Engineering, Shenzhen Campus of Sun Yat-Sen University, Shenzhen 518107, China; renxb@mail2.sysu.edu.cn (X.R.); lilh65@mail.sysu.edu.cn (L.L.)

**Keywords:** power transmission line, small object detection, UAV images, frequency–spatial fusion

## Abstract

Optical Fiber Composite Overhead Ground Wire (OPGW) cables serve dual functions in power systems, lightning protection and critical communication infrastructure for real-time grid monitoring. Accurate OPGW identification during UAV inspections is essential to prevent miscuts and maintain power-communication functionality. However, detecting small, twisted OPGW segments among visually similar ground wires is challenging, particularly given the computational and energy constraints of edge-based UAV platforms. We propose OPGW-DETR, a lightweight detector based on the D-FINE framework, optimized for low-power operation to enable reliable detection. The model incorporates two key innovations: multi-scale convolutional global average pooling (MC-GAP), which fuses spatial features across multiple receptive fields and integrates spectrally motivated features for enhanced fine-grained representation, and a hybrid gating mechanism that dynamically balances global and spatial features while preserving original information through residual connections. By enabling real-time inference with minimal energy consumption, OPGW-DETR addresses UAV battery and bandwidth limitations while ensuring continuous detection capability. Evaluated on a custom OPGW dataset, the S-scale model achieves 3.9% improvement in average precision (AP) and 2.5% improvement in AP_50_ over the baseline. By mitigating misidentification risks, these gains improve communication reliability. As a result, uninterrupted grid monitoring becomes feasible in low-power UAV inspection scenarios, where accurate detection is essential to ensure communication integrity and safeguard the power grid.

## 1. Introduction

With the modernization of power systems, Optical Fiber Composite Overhead Ground Wire (OPGW) has played a crucial role in enhancing grid communication capabilities and enabling remote monitoring and control [1]. This novel cable design, installed on transmission and distribution lines, protects optical fibers from environmental conditions, such as lightning, short circuits, and load variations, thereby ensuring reliability and service life. Due to its wide applicability across various power lines, ease of installation, strong resistance to environmental interference, low line fault rate, and long lifespan, OPGW has become a preferred communication medium extensively utilized within power systems.

Regular inspection and patrol are key steps for ensuring the long-term operational stability of OPGW [1]. These measures not only facilitate early detection of potential issues but also allow preventive actions before problems escalate, thereby minimizing system failures and maintenance downtime. Among these, Unmanned Aerial Vehicle (UAV)-based automated inspection technology is a critical approach to improving patrol efficiency and accuracy. It should be emphasized that the goal of OPGW detection in this work is not to enable physical interaction, such as collision avoidance or robotic manipulation, but rather to support accurate asset documentation and condition assessment from UAV imagery. Specifically, precise identification of OPGW cables facilitates rapid damage localization after natural disasters (e.g., typhoons or ice storms), enables up-to-date inventory management during grid upgrades, and underpins preventive maintenance by revealing early signs of wear or faulty installation. Given these operational requirements, our approach prioritizes reliable visual discrimination between OPGW and visually similar ground wires using only standard RGB cameras, which is a practical choice aligned with existing UAV inspection workflows. However, OPGW cables in UAV imagery appear small-sized, often occluded by complex background elements, such as trees, towers, and conventional ground wires. Aerial inspection of OPGW cables presents several domain-specific computer vision challenges beyond generic small-object detection. First, specular reflections from metallic hardware under direct sunlight frequently saturate local image regions, obscuring shape and texture cues of the heart-shaped ring fitting. Second, atmospheric degradation (e.g., haze, rain) and UAV motion artifacts, including rolling shutter distortion and wind-induced cable vibration, further degrade image quality and geometric consistency. Third, the target exhibits extreme scale variation due to variable flight altitude and viewing angle, while being surrounded by semantically similar distractors such as phase conductors, tower bolts, and insulator strings that create high visual clutter. Finally, operational constraints limit viewpoint diversity, often resulting in foreshortened or partially self-occluded views of the fitting. These factors collectively demand a detector that is not only sensitive to small structures but also robust to illumination extremes, motion degradation, and contextual ambiguity. This makes accurate feature extraction of the target extremely challenging, imposing significant difficulties on detection tasks.

In recent years, the Detection Transformer (DETR) and its variants based on the Transformer architecture have achieved remarkable progress in object detection by eliminating the cumbersome anchor-based design of traditional methods, enabling an end-to-end efficient detection pipeline [2]. Nonetheless, DETR series models generally demand substantial computational resources and demonstrate limited performance when detecting small objects within complex backgrounds, thus struggling to fully meet the stringent requirements of lightweight, high-accuracy, and real-time operation demanded by UAV inspection tasks. Addressing this bottleneck, D-FINE [3], a lightweight DETR variant, achieves faster convergence and reduced computational overhead by redesigning the bounding box regression mechanism and introducing a self-distillation strategy. However, its capabilities in fine-grained feature enhancement and extraction of complex texture information can be further improved.

Therefore, this paper proposes an improved model, termed OPGW-DETR, for detecting OPGW cables and conventional ground wires based on the D-FINE framework. We innovatively introduce a multi-scale convolution global average pooling (MC-GAP) module that integrates spatial features with frequency domain information across multiple receptive fields, significantly enhancing the representation of small targets. Concurrently, a learnable gating mechanism is designed to dynamically balance the fusion of frequency-selective and spatial-domain features, thereby improving the robustness and discriminative power of the model’s feature representations. Experimental results demonstrate that OPGW-DETR significantly improves detection accuracy of OPGW cables and ground wires in UAV images while maintaining lightweight computational resource consumption and low power requirements, effectively meeting the real-time inspection demands of practical transmission lines. Our contributions are summarized as follows:

- MC-GAP for Small-Target Enhancement: We develop an MC-GAP module that aggregates features from different receptive fields via multi-scale convolutions, followed by concatenation, fusion, and global average pooling augmented with spectrally motivated features. By jointly exploiting spatial and frequency cues in a multi-scale manner, MC-GAP strengthens the representation of fine textures and global context for small OPGW targets, leading to notably improved detection accuracy under complex backgrounds.
- Hybrid Gating for Frequency–Spatial Feature Balancing: We design a hybrid gating mechanism that combines global learnable scalars (α, β) with spatial-adaptive gate maps to dynamically weight frequency-enhanced and spatial-enhanced features. The global scalars provide coarse-grained balance control, while the gate maps enable pixel-wise adaptivity. Together with residual connections that preserve the original feature information, this scheme enriches feature diversity and robustness, and alleviates the limitations of conventional convolutional layers constrained by single receptive fields and limited feature expressiveness.

## 2. Related Work

### 2.1. Transmission Line Inspection Technologies

Traditional inspection methods rely on field personnel carrying auxiliary equipment to conduct close-range patrols, enabling timely detection of component defects and potential hazards [4]. The advantage of manual inspection lies in its detailed and intuitive detection capability, which is especially suitable for complex terrain and remote areas. However, its drawbacks include low inspection efficiency, unstandardized inspection data, and difficulties in achieving real-time supervision [5].

To improve inspection efficiency and safety, helicopter-based inspection technology has been gradually adopted. Equipped with high-resolution visible light and infrared thermal imaging devices, helicopters enable long-distance inspection of conductor connectors, insulators, and fittings [6]. Nevertheless, helicopter inspection faces several limitations: susceptibility to weather and airspace restrictions, limited flight duration, difficulty accessing obstructed areas and tower bases, and high operational costs.

Subsequently, owing to their agility and lower costs, UAVs have been applied for intelligent transmission line inspection. UAVs carry high-definition cameras, multispectral sensors, thermal imaging, and LiDAR systems, enabling the acquisition of high-resolution images and 3D point cloud data [6]. However, UAV inspection images often contain complex background information, and transmission line targets, such as optical cables and insulators, are slender structures prone to occlusion. This poses significant challenges to traditional image processing and recognition methods. While offloading image analysis to ground stations or cloud servers is feasible in well-connected environments, real-world power line inspections often occur in remote or mountainous regions with limited or no cellular coverage [7,8]. In such settings, reliance on continuous uplink for image transmission is unreliable and introduces latency that hinders adaptive inspection workflows (e.g., on-the-fly repositioning for detailed joint assessment). Moreover, transmitting high-resolution imagery consumes significant energy and bandwidth, which are critical constraints for battery-powered UAVs operating over long distances [9,10]. To ensure robustness and operational flexibility across diverse deployment conditions, we prioritize a lightweight detection architecture. This design enables real-time inference directly on the UAV when connectivity is poor, while remaining compatible with centralized processing when high-bandwidth links are available.

### 2.2. Learning-Based Object Detection

Deep Convolutional Neural Networks (CNNs) have laid the foundation for object detection [11]. The main CNN-based detection frameworks can be categorized into two-stage and single-stage approaches. Two-stage detectors, exemplified by R-CNN and its variants, use selective search to generate candidate regions, followed by CNN-based feature extraction, classification, and bounding box regression for each region [12]. Fast R-CNN improved speed by sharing convolutional computations, and Faster R-CNN [13] introduced a Region Proposal Network (RPN) to enable end-to-end training, significantly boosting detection efficiency and accuracy. Single-stage detectors like the YOLO series [14] and SSD [15] employ a single neural network to directly predict class probabilities and bounding boxes on the image, achieving end-to-end and efficient inference. YOLOv3 [16] and later versions enhance small object recognition through multi-scale prediction, while RetinaNet [17] introduces focal loss to effectively address class imbalance.

Nevertheless, small and densely packed targets, large scale variations, and complex backgrounds in UAV inspection images present unique challenges to CNNs. To mitigate these issues, Feature Pyramid Networks (FPN) [18] were proposed to enhance multi-scale feature representation. Further improvements, such as Path Aggregation Networks (PANet) [19] and Bidirectional Feature Pyramid Networks (BiFPN) [20], strengthen cross-level feature fusion, enhancing the model’s adaptability to complex scenes. Despite these advances, the intrinsic limited receptive field of CNNs hinders their ability to capture global contextual information adequately.

Transformers, with their powerful self-attention mechanisms and global information modeling capabilities, overcome the limited receptive field issue inherent in CNNs. Carion et al. [2] proposed DETR, which applied a standard Transformer for object detection, realizing truly end-to-end detection by discarding traditional anchor designs and complex post-processing, such as non-maximum suppression. However, the original DETR suffered from slow convergence, requiring approximately 500 training epochs to achieve satisfactory accuracy, and limited context acquisition, which hindered performance on small objects.

To address these drawbacks, Deformable DETR [21] introduced deformable attention to perform sparse spatial sampling, reducing model complexity. Conditional DETR [22] learned conditional spatial queries from decoder embeddings, enabling multi-head cross-attention in the decoder. DAB-DETR [23] optimized query design with dynamic anchor boxes to accelerate convergence. Anchor DETR [24] encoded anchor points as object queries and designed attention variants, improving prediction accuracy and memory efficiency. Efficient DETR [25] proposed a simplified and efficient end-to-end detection pipeline further optimizing convergence speed. Additionally, slow convergence was linked to the discrete bipartite matching component, which is unstable in early training due to randomized optimization. Thus, denoising training strategies and deformable attention in decoder layers were introduced in DN-DETR [26] and DINO [27] to achieve faster convergence.

For real-time detection, RT-DETR [28] designed an efficient hybrid encoder and a minimum uncertainty query selection strategy to fuse multi-scale features and enhance initial query quality, becoming the first real-time end-to-end DETR detector. However, RT-DETR’s limited dynamic perception hindered its ability to detect complex inputs effectively. In response, Dynamic DETR [29] incorporated adaptive dilation and deformable modules in the backbone stage along with dynamic upsampling operators in the neck stage to optimize perceptual capabilities. Moreover, to solve degradation caused by repeated downsampling and poor small object detection, UAV-DETR [30] designed modules to enhance feature perception, semantic representation, and spatial alignment, improving feature expressiveness. D-FINE [3] redefined bounding box regression and introduced an effective self-distillation strategy to optimize prediction methods and enable early model refinement, boosting overall performance. Most DETR models are limited during training by the single-object to single-positive-sample matching scheme, leading to sparse supervision signals that impact training efficiency and detection accuracy. To this end, DEIM-DETR [31] proposed a dense positive sample matching strategy and a novel matching-aware loss function, increasing sample diversity and encouraging the model to focus on high-quality matches.

Compared with the above DETR variants, our work focuses on the unique challenges of small and thin OPGW cables in UAV transmission-line inspection images, where the heart-shaped ring fittings are composed of smooth metallic surfaces and exhibit weak intrinsic texture, offering limited discriminative visual features, particularly in long-range UAV imagery. Building on the lightweight and real-time D-FINE framework, we introduce a frequency–spatial feature enhancement paradigm rather than redesigning the detection head or matching strategy. Specifically, we develop an MC-GAP module that jointly aggregates multi-scale spatial features and spectrally motivated features to strengthen fine-grained texture and global context representation for small targets. In addition, we design a hybrid gating mechanism that dynamically balances frequency-enhanced and spatial-enhanced features in a globally guided yet spatially adaptive manner, effectively mitigating the limited dynamic perception of existing real-time DETR models. These designs are tailored to the characteristics of OPGW targets and UAV inspection scenarios, and thus complement prior DETR improvements that mainly emphasize convergence speed, query design, or generic small-object detection.

While recent advances in attention mechanisms, such as Squeeze-and-Excitation (SE) [32], CBAM [33], and ECA [34], have demonstrated remarkable success in enhancing feature representation through channel or spatial recalibration, these methods primarily operate within the spatial domain and do not explicitly account for frequency characteristics. Similarly, multi-scale convolutional designs like Inception [35] or Res2Net [36] enrich receptive field diversity but treat all frequency components uniformly, often amplifying high-frequency noise that can degrade small-object detection under cluttered backgrounds.

In contrast, our work is motivated by the observation that slender OPGW cables in UAV imagery exhibit distinct spectral properties: their structural integrity is encoded in low-frequency components, while fine textures and environmental interference manifest as high-frequency signals. Rather than learning attention weights over channels or spatial locations, we propose a frequency–spatial collaborative enhancement strategy that explicitly decomposes features into approximate low-frequency (global structure) and multi-scale spatial (local detail) representations. This is achieved through a lightweight MC-GAP module, which leverages the low-pass filtering effect of global average pooling to extract structural context, followed by a hybrid gating mechanism that dynamically balances these complementary streams. Our approach does not aim to replace existing attention modules, but rather to complement them with a frequency-aware perspective tailored to the unique challenges of aerial power line inspection.

## 3. OPGW-DETR with Frequency-Selective Spatial Feature Enhancement for UAV Inspection Images

**Figure 1:**
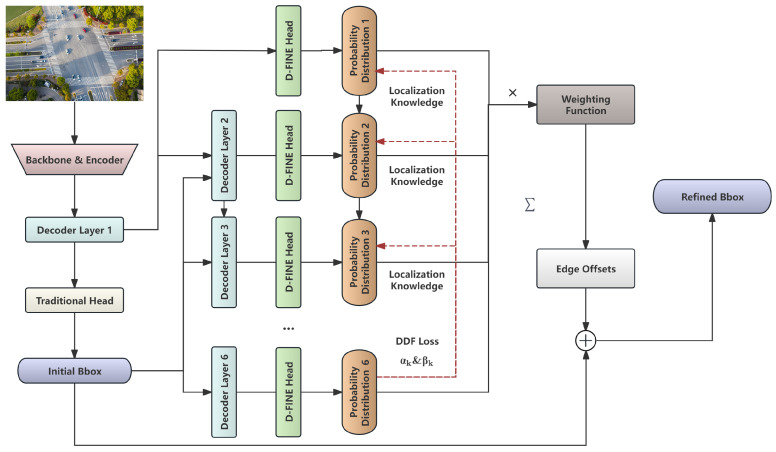
Architecture of D-FINE Model.

D-FINE represents an example of a lightweight real-time DETR design. To improve detection accuracy of RT-DETR, D-FINE introduces fine-grained distribution refinement and globally optimal localization self-distillation components. This design redefines the bounding box regression task, enhances fine-grained intermediate representations, and encourages the model to learn better early adjustments, achieving a balance between speed and high accuracy. These innovations allow RT-DETR to leverage its non-post-processing operations better, adapting more effectively to real-time detection scenarios. The D-FINE model architecture is illustrated in Figure 1.

**Figure 2:**
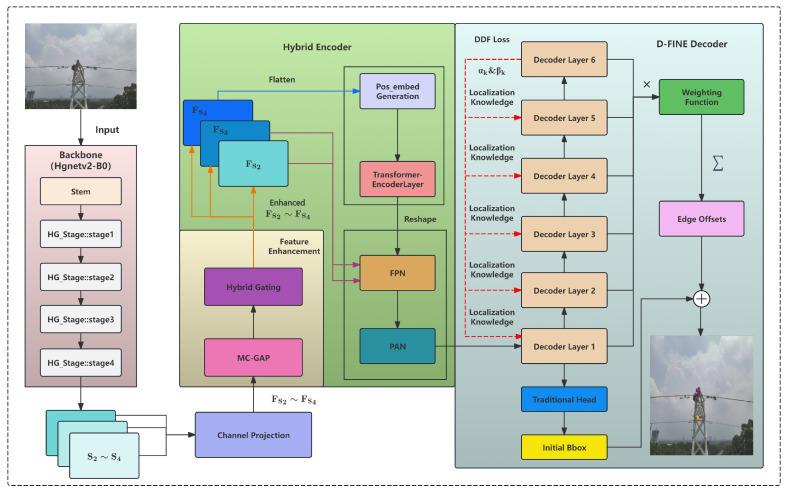
Architecture of OPGW-DETR Model.

OPGW optical cables in power transmission lines captured by UAVs present practical challenges characterized by elongated, twisted shapes and frequent interference from complex backgrounds and occlusions. In response to these challenges, this paper proposes an improved detection model, OPGW-DETR, based on the D-FINE framework. The architecture of the model is illustrated in Figure 2. The model emphasizes enhanced multi-scale perception of small-object features and the auxiliary utilization of spectrally motivated features, thereby enriching feature representation and discriminative capability. These improvements aim to meet the real-time and high-precision detection requirements inherent to UAV inspections. It is important to clarify that our detection target is not the elongated cable itself, but the slender installed at OPGW cable joints. These mechanical components are uniquely present on OPGW cables and serve as a definitive visual marker to distinguish them from conventional ground wires, which lack such fittings. Given their compact, localized structure, typically occupying only 0.08–0.5% of the image area, these fittings behave as small objects rather than linear entities, making bounding box detection a natural and efficient choice. Moreover, our task requires only the presence or absence judgment of such fittings for cable-type classification, not pixel-accurate shape modeling. Bounding boxes thus provide sufficient spatial localization while offering significant advantages in computational efficiency, annotation cost, and deployment feasibility on resource-constrained UAV platforms, which is critical factors for large-scale, real-world power line inspection. Consequently, the term “slender” or “elongated object detection” in this work refers to the challenge of detecting these small, low-contrast fittings within complex aerial scenes, rather than modeling the cable’s linear geometry. Moreover, our system operates within a Level 2 (Partial Autonomy) UAV framework. In this paradigm, flight trajectories are pre-programmed by human operators based on known transmission line coordinates, and the UAV autonomously executes takeoff, waypoint navigation, and landing using GPS/INS guidance. However, continuous operator supervision is required, and no autonomous decision-making, such as dynamic path replanning or obstacle avoidance, is implemented. The proposed OPGW-DETR model functions exclusively as a perception module: it performs real-time detection and classification of heart-shaped ring fittings during flight but does not influence vehicle control or mission logic. Detection results are intended to support post-mission analysis or provide auxiliary visual cues to the operator, ensuring alignment with current safety and regulatory practices in power infrastructure inspection.

To provide a comprehensive understanding of our data collection methodology, we detail the specifications of our imaging system, operational flight parameters, data acquisition protocol, and curation and annotation pipeline. Our UAV-mounted camera system is equipped with an RGB sensor offering 20-megapixel resolution. The focal length is set to 24 mm equivalent, providing a wide-angle configuration that captures sufficient context around the transmission towers. Images are stored in JPEG format with automatic exposure compensation to ensure consistent lighting conditions across different environments. Operational flights were conducted with strict adherence to predefined parameters. The UAVs flew at altitudes ranging from 30 to 50 m above ground level (AGL) and maintained velocities between 3 to 5 m per second during image acquisition. Control was manual, performed by certified operators who could make real-time adjustments based on visual feedback. This approach enabled us to optimize the quality of each captured image by dynamically adjusting angles and distances to minimize occlusions and enhance target visibility. The dataset was collected over a three-year period, focusing on suburban and low-density urban environments. Capture sessions were scheduled during morning and afternoon hours to take advantage of favorable meteorological conditions. We specifically avoided adverse weather such as rain, fog, or strong winds to ensure adequate illumination and minimize motion blur. Certified operators made real-time decisions about shooting angles and distances, which helped mitigate potential biases compared to post-capture video frame extraction methods. All images were directly captured rather than extracted from continuous video streams, ensuring intentional high-quality framing and minimizing redundancy. Following the initial capture, raw imagery underwent a two-stage processing workflow. In the first stage, images lacking OPGW cables or conventional ground wires were excluded. The remaining images were then manually annotated by domain experts using standardized bounding box annotations. These experts delineated target categories and spatial coordinates, providing precise labels for model training and evaluation. This rigorous curation process ensured that the final dataset accurately reflected the challenges faced in real-world power-line inspections while maintaining high data quality and minimizing unintended acquisition biases.

**Figure 3:**
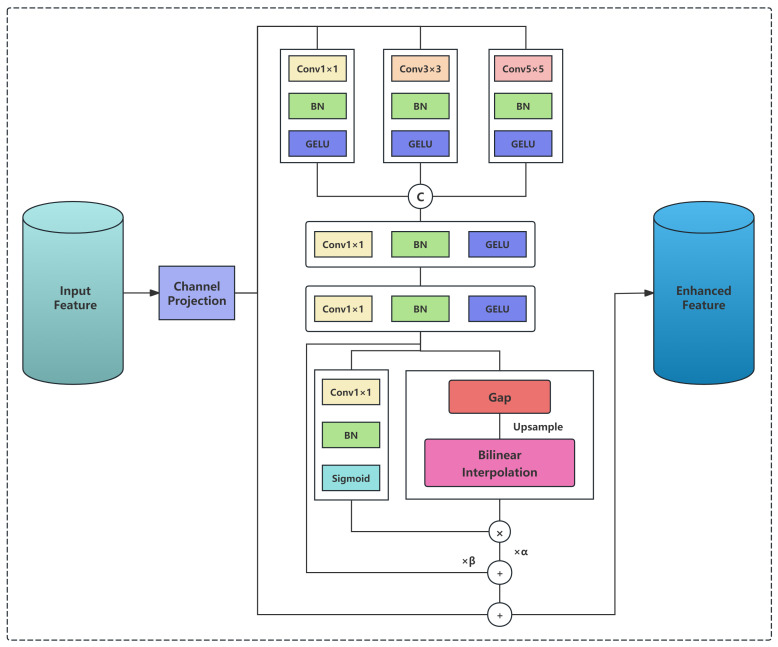
MC-GAP Architecture with Integrated Gating Mechanism.

The overall framework inherits the lightweight and efficient Transformer-based detection architecture of D-FINE and introduces a novel MC-GAP feature enhancement module, as depicted in Figure 3. This module captures rich spatial receptive field features through parallel convolutions with multiple kernel sizes and employs global average pooling as an effective low-frequency information aggregation method to supplement the global semantic context of spatial features. Subsequently, a hybrid gating mechanism is designed to dynamically fuse spatial and low-frequency domain information, improving robustness in recognizing targets with intricate details. Meanwhile, residual connections preserve the original feature information to prevent degradation.

Targeting the challenges posed by the small size, diverse morphology, and complex backgrounds of cable targets seen from UAV perspectives, this architecture effectively integrates the complementary advantages of spatial and frequency features. It enhances the model’s fine-grained representation of curved, slender while balancing real-time inference efficiency with detection accuracy.

### 3.1. Multi-Scale Convolution-Based Global Average Pooling (MC-GAP)

The model takes an RGB input image of size 3×H×W. The backbone network employs the pre-trained HGNetv2 to extract feature maps from *N* stages, where *N* varies with model scale. In this paper, we focus on stages i∈{2,3,4}, which output multi-scale feature maps $X_{S_{i}}\in R^{B\times C_{i}\times \frac{H}{s_{i}}\times \frac{W}{s_{i}}},i=2,3,4$, where *B* denotes the batch size, $C_{i}$ represents the number of channels at stage *i*, and $s_{i}$ is the downsampling factor. These multi-stage feature maps capture hierarchical information from fine details to high-level semantics.

To unify the feature dimensions across different stages and facilitate subsequent multi-scale fusion and encoder processing, three independent 1×1 convolution layers with BatchNorm modules are employed to project all feature maps to a unified hidden dimension *D*:

(1) $$F_{S_{i}}=\mathrm{BN}\left({\mathrm{Conv}}_{1\times 1}\left(X_{S_{i}}\right)\right),\;i=2,3,4,$$

yielding projected features $F_{S_{i}}\in R^{B\times D\times \frac{H}{s_{i}}\times \frac{W}{s_{i}}}$, where *D* is the unified channel dimension, which varies with the model scales.

#### 3.1.1. Multi-Scale Parallel Convolution Processing

The MC-GAP module operates in scenarios where the target object has minimal texture, yet is embedded within a texture-rich background (e.g., transmission towers, vegetation, and sky). It adaptively enhances structural and contextual cues to compensate for the target’s low textural complexity. To effectively capture these characteristics, the MC-GAP module performs uniform enhancement across stages $S_{2}$, $S_{3}$, and $S_{4}$ after feature projection on the *N* extracted feature maps $F_{S_{i}}$ (i=2,3,4).

- Step 1: Parallel Multi-scale Convolution. Three convolutional layers with kernel sizes 1×1, 3×3, and 5×5 are applied in parallel to extract multi-scale spatial features. All convolutions include Batch Normalization and GELU activation:
  (2) $$F_{S_{i}}^{\left(k\right)}=\mathrm{GELU}\left(\mathrm{BN}\left({\mathrm{Conv}}_{k\times k}\left(F_{S_{i}}\right)\right)\right),\;k\in \left\{1,3,5\right\}.$$
  The 1×1 convolution captures point-wise information, the 3×3 convolution aggregates local fine textures enhancing the cable’s fibrous details, and the 5×5 convolution integrates semantic and textural continuity over a larger spatial range.
- Step 2: Channel Concatenation. The three branches are concatenated along the channel dimension:
  (3) $$F_{\mathrm{concat}}^{\left(i\right)}=\mathrm{Concat}\left(F_{S_{i}}^{\left(1\right)},F_{S_{i}}^{\left(3\right)},F_{S_{i}}^{\left(5\right)}\right),$$
  yielding $F_{\mathrm{concat}}^{\left(i\right)}\in R^{B\times 3D\times \frac{H}{s_{i}}\times \frac{W}{s_{i}}}$.
- Step 3: Feature Fusion. A 1×1 convolution is used to fuse and reduce the dimensionality back to *D* channels:
  (4) $$F_{\mathrm{fused}}^{\left(i\right)}=\mathrm{GELU}\left(\mathrm{BN}\left({\mathrm{Conv}}_{1\times 1}\left(F_{\mathrm{concat}}^{\left(i\right)}\right)\right)\right),$$
  satisfying $F_{\mathrm{fused}}^{\left(i\right)}\in R^{B\times D\times \frac{H}{s_{i}}\times \frac{W}{s_{i}}}$.

This parallel multi-scale convolution design enables the model to simultaneously capture spatial features at different receptive field scales, which is particularly important for detecting cable targets with varying appearances and sizes.

#### 3.1.2. Approximate Extraction of Low-Frequency Information

Traditional frequency domain processing methods (e.g., FFT [37]) are computationally expensive and significantly impact real-time performance. This paper employs global average pooling (GAP) [38] as an efficient alternative for extracting approximate low-frequency information.

For the fused feature $F_{\mathrm{fused}}^{\left(i\right)}\in R^{B\times D\times \frac{H}{s_{i}}\times \frac{W}{s_{i}}}$, we first apply a 1×1 convolution for feature adjustment:

(5) $$F_{\mathrm{adj}}^{\left(i\right)}=\mathrm{GELU}\left(\mathrm{BN}\left({\mathrm{Conv}}_{1\times 1}\left(F_{\mathrm{fused}}^{\left(i\right)}\right)\right)\right).$$

Subsequently, global average pooling is performed to extract approximate low-frequency information:

(6) $$F_{\mathrm{gap}}^{\left(i\right)}=\mathrm{GAP}\left(F_{\mathrm{adj}}^{\left(i\right)}\right)=\frac{1}{HW}\sum_{h=1}^{H/s_{i}}\sum_{w=1}^{W/s_{i}}F_{\mathrm{adj}}^{\left(i\right)}\left[:,:,h,w\right],$$

where $F_{\mathrm{gap}}^{\left(i\right)}\in R^{B\times D\times 1\times 1}$ and GAP denotes the global average pooling operation. From a frequency domain perspective, this spatial smoothing operation acts as a low-pass filter that effectively suppresses high-frequency components (detail noise and abrupt changes) while preserving low-frequency components (overall structure and global texture information) of the features.

Finally, bilinear interpolation upsampling is used to restore the pooled result to the original spatial size:

(7) $$F_{\mathrm{freq}}^{\left(i\right)}={\mathrm{Upsample}}_{\mathrm{bilinear}}\left(F_{\mathrm{gap}}^{\left(i\right)},\mathrm{size}=\left(\frac{H}{s_{i}},\frac{W}{s_{i}}\right)\right)\in R^{B\times D\times \frac{H}{s_{i}}\times \frac{W}{s_{i}}}.$$

This achieves spatial alignment between the low-frequency enhanced features and spatial features for subsequent fusion.

### 3.2. Hybrid Gating Mechanism and Residual Connection

**Figure 4:**
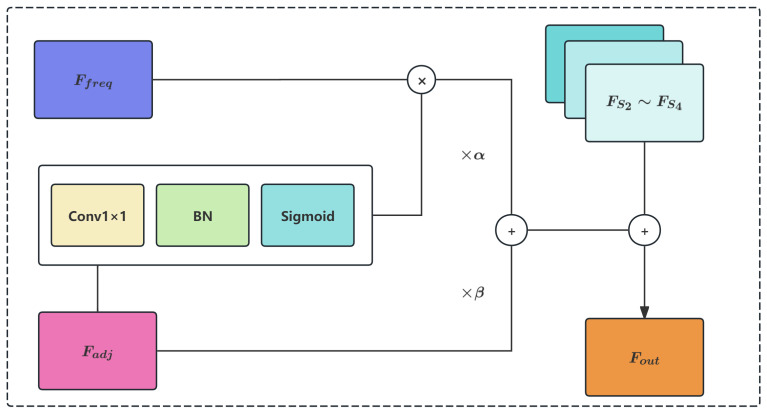
Hybrid Gating Mechanism for Frequency–Spatial Balancing.

To adaptively balance the contributions of spatially fused features and frequency domain low-frequency information, this paper introduces a hybrid learnable gating mechanism that combines global balance control with spatial adaptivity. The structure is illustrated in Figure 4.

First, a 1×1 convolution followed by batch normalization and Sigmoid activation generates a spatial-adaptive gate map:

(8) $${\mathrm{Gate}}^{\left(i\right)}=\sigma \left(\mathrm{BN}\left({\mathrm{Conv}}_{1\times 1}\left(F_{\mathrm{fused}}^{\left(i\right)}\right)\right)\right)\in R^{B\times D\times \frac{H}{s_{i}}\times \frac{W}{s_{i}}},$$

where σ(·) denotes the Sigmoid function that constrains values to the range (0,1), and BN(·) represents batch normalization. Meanwhile, two global learnable scalar parameters α,β∈R are introduced to control the overall contribution balance, initialized as $\alpha_{0}=0.1,\beta_{0}=0.9$. Unlike traditional complementary gating mechanisms where β=1−α, our design allows α and β to be learned independently, providing greater flexibility in balancing frequency and spatial information contributions.

This hybrid mechanism enables adaptive fusion strategy adjustment according to both global statistics and local spatial content:

- Spatial Adaptivity: The ${\mathrm{Gate}}^{\left(i\right)}$ map varies across spatial locations, enabling the model to emphasize frequency features in regions where global structural information is crucial while preserving spatial details where fine textures dominate;
- Global Balance: The scalar parameters α and β provide coarse-grained control over the relative importance of frequency versus spatial pathways across the entire feature map, allowing the model to learn task-specific optimal weighting strategies.

Finally, the enhanced output feature is obtained through hybrid weighted fusion and residual connection:

(9) $$F_{\mathrm{out}}=F_{S_{i}}+\alpha \cdot \left({\mathrm{Gate}}^{\left(i\right)}⊙F_{\mathrm{freq}}^{\left(i\right)}\right)+\beta \cdot F_{\mathrm{fused}}^{\left(i\right)},$$

where ⊙ denotes element-wise (Hadamard) product and · represents scalar multiplication with broadcasting. Due to the associativity of multiplication, $\alpha \cdot ({\mathrm{Gate}}^{\left(i\right)}⊙F_{\mathrm{freq}}^{\left(i\right)})$ is equivalent to $\left(\alpha \cdot {\mathrm{Gate}}^{\left(i\right)}\right)⊙F_{\mathrm{freq}}^{\left(i\right)}$. The residual connection (adding back $F_{S_{i}}$) preserves the complete information of the original projected features, preventing network degradation, gradient vanishing, and training instability issues.

After processing through the MC-GAP module, spatial domain features and low-frequency enhanced features (obtained via the low-pass filtering effect of GAP) achieve effective cooperative enhancement through the hybrid gating mechanism, significantly improving the model’s representation capability for small twisted cable targets and their contextual environment. This frequency–spatial collaborative enhancement mechanism improves the hierarchical representation, discriminability, and robustness against high-frequency noise interference.

## 4. Numerical Results

### 4.1. Experimental Setup

We collected a total of 3137 images of two types of transmission towers captured by UAVs under different environments and angles. Manual annotation was performed using LabelImg. Among these, the OPGW optical cable of the steel towers is labeled as O1, the ordinary ground wire as D1; the OPGW cable of the straight-line towers is labeled as O2, and the ordinary ground wire as D2. The annotated dataset is saved in COCO format and split into training, validation, and test sets with a ratio of 3:1:1.

To ensure transparency and facilitate reproducible research, we provide a detailed quantitative characterization of our dataset beyond the total image count and resolution. The dataset contains 6344 annotated instances across four categories: OPGW cables (O1, O2) and conventional ground wires (D1, D2). As shown in Table 1, OPGW classes constitute 53.0% of all labels (3365 instances), while ground wires account for 47.0% (2979 instances). The class distribution is balanced (largest-to-smallest ratio: 2.49:1), and the splits across training, validation, and test sets exhibit high consistency (standard deviation < 0.8%). Bounding box statistics (Table 2) reveal an extreme scale variation: median width and height are 45.7 px and 7.8 px, respectively, while the coefficient of variation exceeds 115% for all size metrics, indicating highly diverse capture distances. Critically, 74.1% of targets are classified as “tiny” or “small” (<0.5% of image area; Table 3), with only 3.8% exceeding 2% area. This confirms that our dataset emphasizes realistic, high-difficulty inspection scenarios. Spatially, 44.9% of bounding box centers fall within the central third of the image (Table 4), but over 23% appear in corner regions, demonstrating non-trivial off-center coverage. The vertical distribution shows slight asymmetry (more targets in lower half), likely due to UAV shooting angles, while horizontal symmetry is well preserved. Finally, the number of annotated objects per image varies meaningfully (Table 5): 41.0% of images contain a single target (e.g., long-range or partially occluded views), 32.6% show two targets (typical dual-wire setups), and 26.0% feature three or more, capturing complex tower junctions or multi-circuit lines. This diversity reflects real operational conditions and underscores the need for robust detectors capable of handling variable scene complexity. Together, these statistics validate that our dataset presents a challenging yet representative benchmark for UAV-based power-line inspection.

All models were trained on Ubuntu 20.04 LTS systems equipped with NVIDIA (Santa Clara, CA, USA) Quadro RTX 8000 GPUs. The Python version used is 3.11.9, and PyTorch version is 2.8.0+cu126. Our model is based on D-FINE [3], employing HGNetv2 as the backbone consistent with D-FINE. We implemented our proposed method and trained the model for 280 epochs with a batch size of 16. Early stopping was applied, monitoring the metric Bounding Box Average Precision (AP), calculated as the mean AP over 10 IoU thresholds ranging from 0.5 to 0.95. The patience was set to 35 epochs. The OPGW-DETR network is optimized by AdamW [39]. Learning rates were set to 0.0004, 0.0002, 0.0002 and 0.00025 for backbones of sizes N, S, M and X, respectively. Input images are resized to 640×640, and COCO standard metrics are reported, including mAP_50_ and mAP_50∼95_. While mAP is a standard metric for evaluating object detectors on static image datasets, its relevance to real-world UAV inspection warrants clarification. In operational settings, a cable joint need only be detected in one of multiple consecutive frames as the drone passes by; perfect per-frame recall is unnecessary. However, our dataset comprises individually labeled images without inter-frame correspondence, making video-level or coverage-based metrics inapplicable.

We compare four model variants of different scales, N, S, M, and X, designed for various application scenarios and computational resource constraints. These variants differ in backbone network configuration, feature extraction stages, and channel dimensions to balance detection accuracy with computational efficiency. All variants adopt HGNetv2 as the feature extraction backbone, but utilize pre-trained models of different depths. HGNetv2 comprises 4 stages, with returned stage features controlled by parameter adjustment. The specific configurations are shown in Table 6, where $C_{i}$ denotes the number of output channels at the *i*-th returned stage of the backbone network, $s_{i}$ represents the corresponding downsampling stride, and *D* is the hidden dimension after channel projection.

### 4.2. Detection Performance and Efficiency Comparison

**Figure 5:**
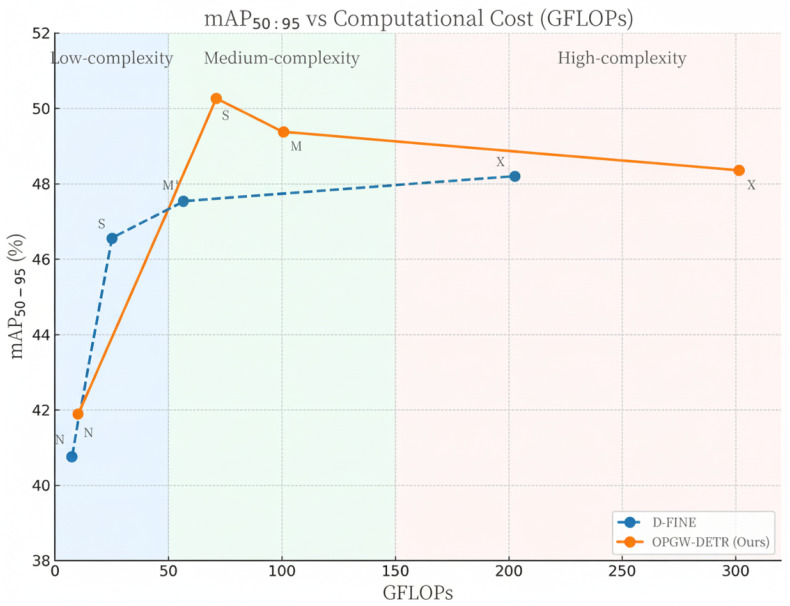
The accuracy-efficiency trade-off.

We present a performance curve to characterize the accuracy–efficiency trade-off in Figure 5, plotting mAP_50∼95_ against computational cost (GFLOPs) across all model variants. This visualization supersedes the previous tabular comparison, which could mislead readers into assuming uniform scaling behavior across architectures. The curve reveals three operational regimes:

- **Low-complexity regime (<50 GFLOPs)**: Here, D-FINE variants achieve higher mAP per GFLOP. Our model’s architectural components (e.g., MC-GAP, hybrid gating) incur fixed overhead that cannot be amortized at very low compute budgets, resulting in suboptimal efficiency.
- **Medium-complexity regime (50–150 GFLOPs)**: OPGW-DETR demonstrates clear superiority. With sufficient capacity, the feature enhancement and attention mechanisms effectively capture discriminative cues from small, cluttered targets, yielding up to +3.9% mAP gain over baselines at comparable GFLOPs.
- **High-complexity regime (>150 GFLOPs)**: Performance gains saturate. Over-parameterized M/X variants show signs of overfitting and degraded gradient flow, limiting further improvements despite increased computation.

Notably, the medium-complexity regime corresponds closely to the computational capabilities of current edge devices used in UAV-based power line inspection (e.g., NVIDIA Jetson AGX Orin). Thus, OPGW-DETR offers meaningful practical advantages where they matter most.

Under the lightweight configuration, OPGW-DETR-S attains the highest accuracy among all compared methods, while still satisfying the stringent constraints of low power consumption and low computational complexity required by real-time embedded platforms (e.g., edge devices deployed on transmission lines or in substations). This balance between accuracy and efficiency is particularly important for long-term online monitoring scenarios, where hardware resources and energy budgets are limited.

While GFLOPs and parameter counts provide initial indicators of computational efficiency, real-world UAV deployment demands hardware-aware validation. We therefore profile GPU power consumption, memory footprint, and utilization under inference conditions in Table 7. Notably, our OPGW-DETR-N consumes only 66 W at idle, which is significantly lower than comparable DETR variants, and maintains a modest 7.6 GB memory footprint, making it suitable for edge platforms such as Jetson AGX Orin. Although peak power during active inference is higher due to attention computation, the low idle power aligns with typical UAV operational patterns involving frequent standby periods. Thus, our energy-efficient design refers not to minimal FLOPs alone, but to a practical balance of accuracy, memory, and power characteristics conducive to aerial inspection.

Furthermore, we compare our model against other lightweight detectors with similar computational budgets in Table 8. Even under roughly comparable FLOPs and parameter counts, our method consistently achieves superior precision, indicating that the proposed architectural design and hybrid frequency–spatial enhancement strategy provide more effective feature utilization than conventional lightweight backbones and detection heads.

### 4.3. Performance Gains from MC-GAP and Hybrid Gating

We conduct ablation studies on the collected OPGW dataset using OPGW-DETR-S to quantify the contribution of each architectural component to the overall detection performance. Table 9 summarizes the results for different model configurations. In this analysis, MC-GAP denotes the multi-scale convolution-based global average pooling feature enhancement module, while Gate represents a hybrid gating mechanism that employs sigmoid activation and batch normalization to adaptively balance the stage-wise enhanced spatial features $F_{S_{2}}$ to $F_{S_{4}}$ with the corresponding approximate low-frequency features.

In the configuration that uses only the MC-GAP module (i.e., without Gate), the enhanced spatial features and low-frequency features $F_{\mathrm{freq}}$ are fused with a fixed equal ratio (α=0.5,β=0.5). This simple, non-learnable fusion ignores image content and scene complexity, and thus cannot fully exploit the complementary nature of frequency–spatial information. In contrast, the hybrid gating mechanism learns content-aware weights, allowing the model to dynamically emphasize spatial details or low-frequency context depending on the local characteristics of twisted cable targets and backgrounds. This is particularly beneficial for complex field environments with varying illumination, background clutter, and cable appearances.

The baseline D-FINE-S achieves 76.0% mAP_50_ and 46.7% mAP_50∼95_. After introducing the MC-GAP module, the detector gains a clear improvement, with mAP_50_ increasing to 78.4% and mAP_50∼95_ rising to 49.4%. These gains demonstrate that explicitly enhancing multi-scale features with approximate low-frequency information is effective for small, elongated OPGW targets that are easily affected by high-frequency noise.

When the gating mechanism is further integrated into MC-GAP, OPGW-DETR-S achieves the best overall performance, reaching 78.5% mAP_50_ and 50.6% mAP_50∼95_. At the same time, the model maintains a computational cost of only 68.9 GFLOPs and 17.2M parameters, indicating that the proposed frequency–spatial hybrid gating introduces negligible overhead relative to the obtained accuracy gains. This balance between performance and efficiency confirms that the OPGW-DETR architecture is well suited for real-time deployment on low-power, resource-constrained embedded platforms, such as edge devices mounted on inspection robots or unmanned aerial vehicles for online transmission line monitoring.

**Figure 6:**
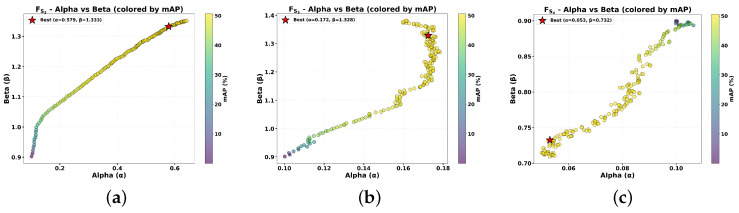
Hybrid Gating Mechanism Parameter Learning Dynamics. (**a**–**c**) Exploration of optimal configurations of parameters α and β for different channel-projected features (representing $F_{S_{2}}$, $F_{S_{3}}$ and $F_{S_{4}}$, respectively) with respect to mAP.

We analyzed the optimal hybrid gating parameter configurations ($\alpha^{*}$, $\beta^{*}$) that achieve peak detection performance. Figure 6a–c visualizes the parameter space, where the color intensity of each point reflects the corresponding mAP, and star markers indicate the optimal configurations. Table 10 summarizes these optimal values.

It can be observed that as the semantic hierarchy ascends, the contribution of frequency-selective enhancement must progressively decrease relative to spatial-domain processing. This hierarchical trade-off reflects the fundamental differences among features at different levels:

- Low-level features ($F_{S_{2}}$) require substantial frequency-selective enhancement ($\alpha^{*}>0.5$) to amplify fine-grained textures and edge information. The relatively balanced ratio indicates that low-level representations benefit nearly equally from both domains. The high absolute values ($\beta^{*}>1.3$) suggest that both frequency and spatial enhancements are necessary to compensate for the inherently limited semantic discriminability of low-level features.
- Mid-level features ($F_{S_{3}}$) exhibit a pronounced shift toward spatial-domain dominance. While maintaining a high $\beta^{*}$, the substantial reduction in $\alpha^{*}$ indicates that mid-level semantic abstractions are susceptible to frequency-selective perturbations. At this level, features encode partially hierarchical patterns and compositional structures, relying on spatial coherence rather than high-frequency details. Excessive frequency enhancement (α>0.2) may introduce artifacts that compromise semantic information acquisition.
- High-level features ($F_{S_{4}}$) demonstrate an extreme spatial bias, with frequency enhancement nearly eliminated. The minimal $\alpha^{*}$ value reflects that high-level semantic representations exhibit minimal dependence on spectrally motivated features while being particularly sensitive to high-frequency noise therein. Notably, $\beta^{*}$ also falls below 1.0, indicating that high-level features obtained from the pretrained backbone already possess sufficient representational capacity, requiring only conservative spatial refinement.

### 4.4. Analysis of Accuracy Improvement and Loss Stabilization in OPGW-DETR

**Figure 7:**
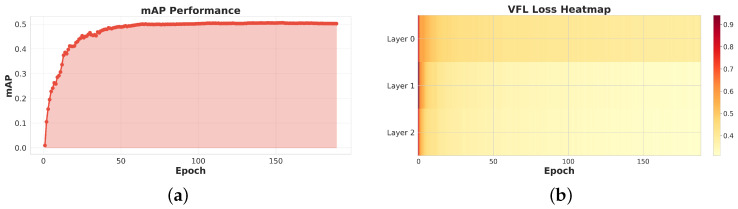
OPGW-DETR-S model training visualization dashboard. (**a**) mAP performance showing model accuracy improvement over training epochs. (**b**) VFL loss heatmap across three decoder layers.

In Figure 7, the mAP curve exhibits a clear two-stage learning behavior. In the first 50 epochs, the model undergoes a rapid learning phase, with mAP increasing from 0.02 to about 0.48, indicating that the backbone and detection head quickly capture the dominant discriminative features of OPGW targets and background. Afterward, training enters a refinement phase in which mAP gradually converges to 0.49–0.50. This slow saturation suggests that the model is performing fine-grained feature calibration, such as improving localization accuracy for partially occluded cables and reducing confusion with visually similar background structures.

**Figure 8:**
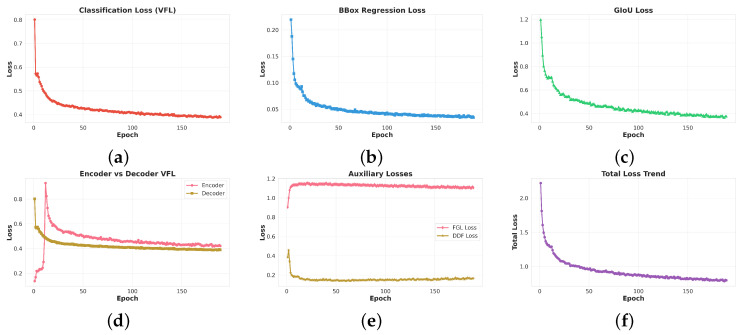
(**a**) Classification loss (VFL) convergence trend. (**b**) BBox regression loss evolution. (**c**) GIoU localization loss trajectory. (**d**) Comparison of Encoder vs Decoder VFL losses. (**e**) Auxiliary losses including FGL and DDF components. (**f**) Total combined loss trend throughout training.

In Figure 8, All primary loss components show stable and consistent convergence, reflecting healthy optimization dynamics. The classification loss (VFL) decreases from 0.80 to 0.40, while the bounding box regression loss drops from 0.22 to 0.03, corresponding to an 86% reduction. Meanwhile, the GIoU loss falls from 1.2 to 0.38. These losses stabilize after approximately epoch 50, indicating that both category discrimination and spatial localization have largely adapted to the data distribution, with no signs of divergence or overfitting. The VFL loss heatmap further reveals relatively uniform learning patterns across the three decoder layers, with all layers exhibiting similar convergence trends. This suggests that the multi-layer decoding structure contributes consistently to classification performance, without any single layer dominating or lagging behind. A comparison between encoder and decoder VFL losses shows similar final values (both around 0.42), but the decoder converges slightly faster in the early epochs. This behavior implies that the decoder benefits more directly from the supervision signal and quickly learns task-specific representations, while the encoder gradually refines the global feature encoding. The total loss decreases from 2.2 to 0.82 (a 63% reduction) and remains stable in the late training stage without noticeable oscillations, indicating that the learning rate schedule and regularization settings are appropriate. The DDF loss converges rapidly from 0.4 to 0.15, demonstrating that the dynamic decomposition features are learned efficiently and do not introduce optimization instability. In contrast, the FGL loss remains stable around 1.15–1.2 throughout training, reflecting its role as a relatively steady regularization or guidance term rather than a sharply decreasing objective. Together, these trends confirm that the proposed OPGW-DETR-S training pipeline is well-conditioned and that both the main detection branch and auxiliary modules contribute effectively to the final performance.

### 4.5. Frequency-Selective Analysis of Feature Enhancement

To rigorously substantiate the frequency-selective nature of our enhancement module, we conduct a comprehensive spectral analysis of intermediate feature maps. All analyses are performed on features extracted after the projection layer in the hybrid encoder (denoted as *Projected*) and after our frequency-selective enhancement module (*UAV-Enhanced*), using multi-scale 2D Fast Fourier Transform (FFT) on 256×256 feature patches from the OPGW validation set.

**Figure 9:**
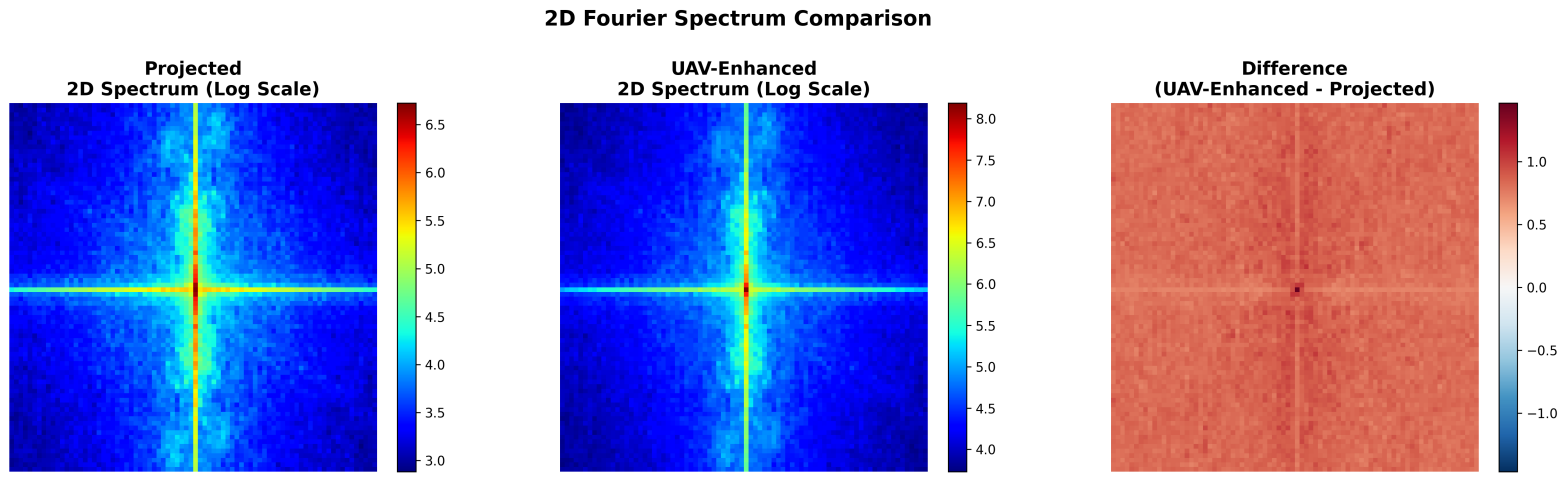
Two-dimensional spectral comparison of feature maps (post-projection in the hybrid encoder). (**Left**): Projected; (**Middle**): UAV-Enhanced; (**Right**): Difference (enhanced minus projected, log scale).

As shown in Figure 9, the magnitude spectra of *Projected* features exhibit dispersed energy with noticeable high-frequency components in peripheral regions, whereas *UAV-Enhanced* features display markedly concentrated energy at the DC component (center), accompanied by a uniformly positive difference map, indicating additive amplification of low frequencies without redistributing existing energy. This pattern is characteristic of low-pass filtering behavior implemented implicitly through spatial operations.

**Figure 10:**
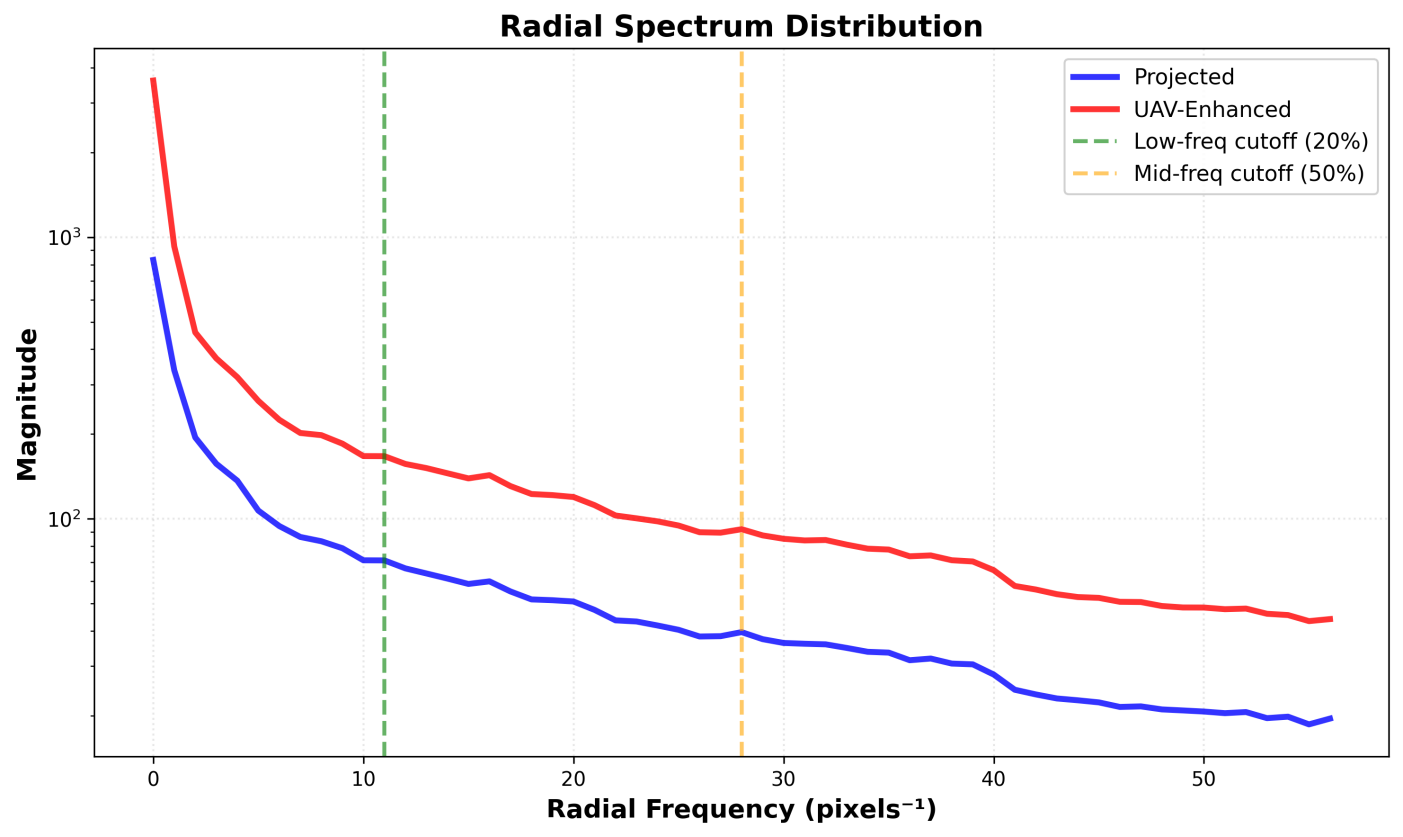
Radial spectral distribution comparison of feature maps.

This observation is quantified in Figure 10 via radial spectral profiles. The enhanced curve (red) consistently exceeds the projected curve (blue) within the low-frequency band (0–$10\;{\mathrm{px}}^{-1}$), while both converge beyond $30\;{\mathrm{px}}^{-1}$, confirming selective low-frequency emphasis.

**Figure 11:**
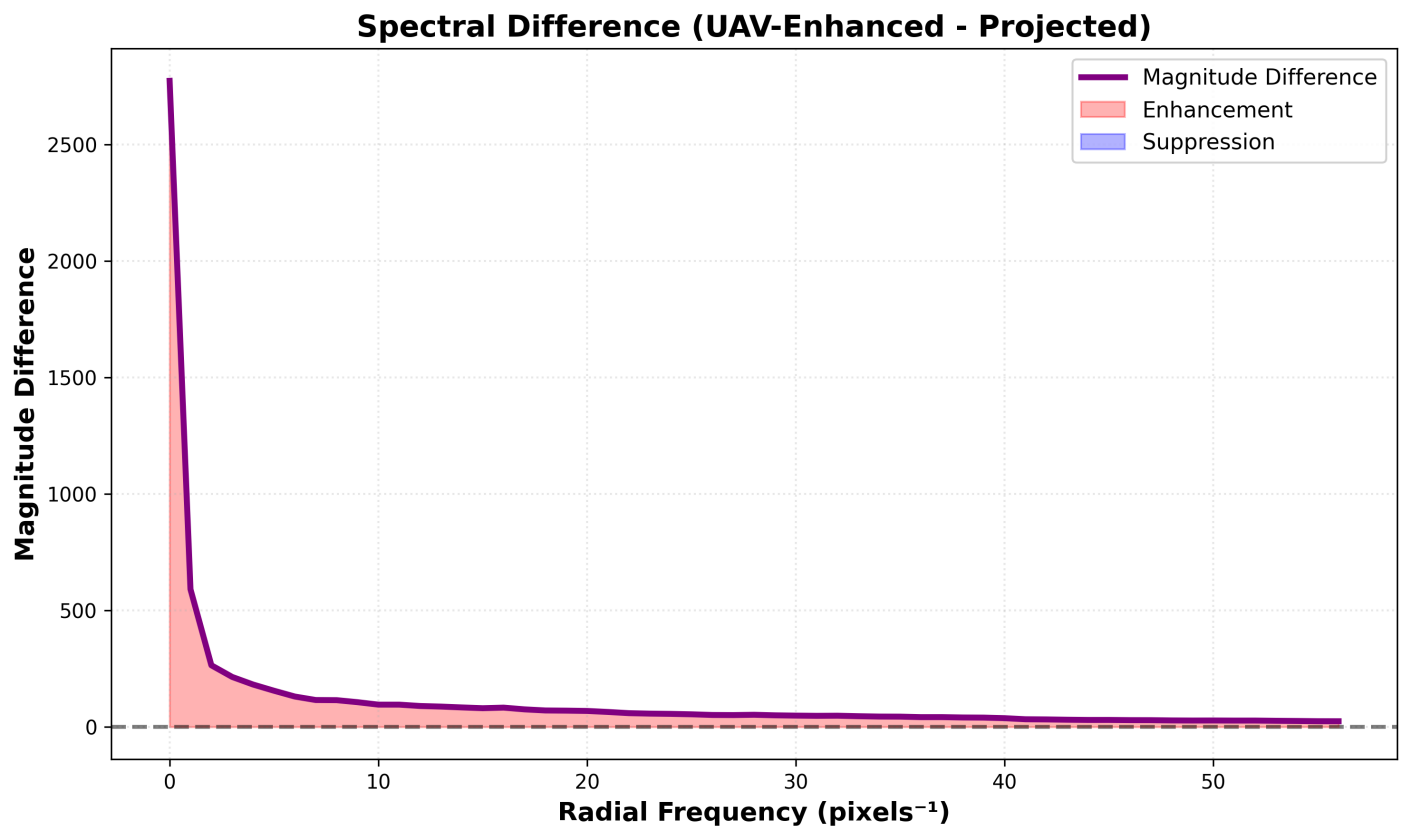
Spectral differences vs. radial frequency.

Figure 11 further decomposes this effect: a sharp peak of +2500 amplitude at 2–$3\;{\mathrm{px}}^{-1}$ reflects strong enhancement of ultra-low frequencies that encode global cable geometry; rapid decay to near-zero by $15\;{\mathrm{px}}^{-1}$ establishes an effective cutoff frequency; and negligible oscillation (±100, <4% of peak) at higher frequencies confirms no active suppression or amplification of mid/high-frequency content. Critically, the absence of negative differences demonstrates that enhancement is purely additive, not competitive.

**Figure 12:**
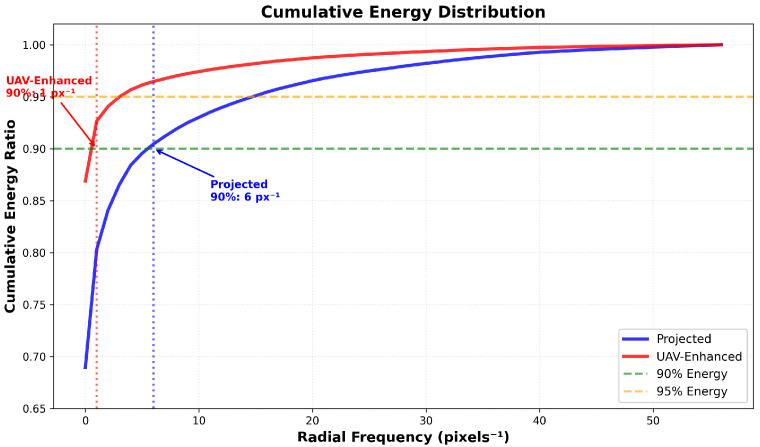
Analysis of cumulative energy distribution vs. radial frequency.

These trends are summarized in Table 11, which reports key frequency-selective metrics:

- Low-frequency energy (0–$10\;{\mathrm{px}}^{-1}$) increases from 93.0% to 97.4% (+4.7% relative);
- High-frequency energy (50–100% Nyquist) drops from 2.2% to 0.8% (–63.6%);
- The spectral centroid shifts leftward from 2.33 to 0.88 ${\mathrm{px}}^{-1}$ (–62.3%);
- The 90% cumulative energy threshold moves from 6 ${\mathrm{px}}^{-1}$ to 1 ${\mathrm{px}}^{-1}$ (83.3% reduction), as visualized in Figure 12.

This dramatic spectral compression implies that post-enhancement features encode the majority of their information in ultra-low frequencies, while attenuating high-frequency clutter from vegetation, tower textures, and sensor noise.

**Figure 13:**
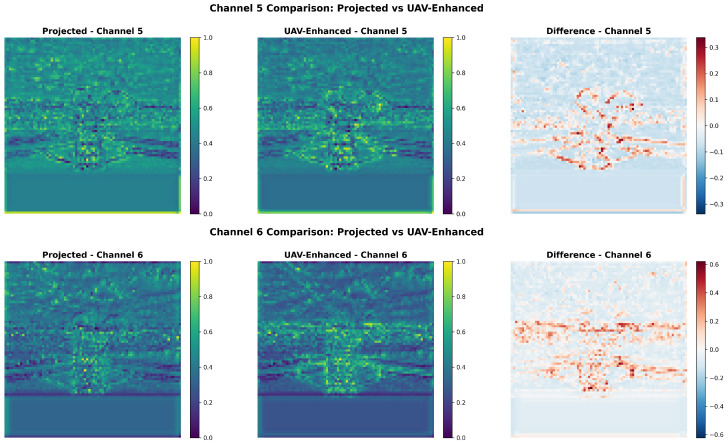
Feature map comparisons for two representative channels. Each row shows (from **left** to **right**): original projected features, enhanced features, and their pixel-wise difference (RdBu colormap).

Finally, Figure 13 provides spatial-domain corroboration. Side-by-side comparisons of two representative channels reveal that *Projected* features contain pervasive high-frequency background noise, whereas *UAV-Enhanced* features exhibit smoother, more coherent responses along cable trajectories. The difference maps (RdBu colormap) show positive deviations (red) aligned with global linear patterns and negative deviations (blue) localized to noisy regions, directly mirroring the low-pass spectral behavior. This dual-domain consistency confirms that our module enhances frequency-selective signal-to-noise ratio (SNR), thereby improving spatial representation fidelity for fine, low-contrast targets.

Collectively, these analyses validate that our frequency-selective enhancement operates without explicit FFT/IFFT transforms, but rather through spatial mechanisms that implicitly emulate low-pass filtering, justifying our revised terminology and supporting the design’s efficacy in UAV-based OPGW detection.

## 5. Discussion on UAV Images Detection with OPGW-DETR

Compared with existing UAV-based power transmission line object detection models, OPGW-DETR exhibits two notable advantages. First, built upon the improved D-FINE framework, OPGW-DETR successfully eliminates the need for complex anchor box design and post-processing steps, achieving an end-to-end efficient detection pipeline. This greatly simplifies model deployment and maintenance complexity. Second, OPGW-DETR introduces the MC-GAP feature enhancement module and designs a learnable gating mechanism to dynamically balance and fuse spatial domain and low-frequency domain features. As demonstrated in Table 7 and Table 8, our model attains favorable detection accuracy while maintaining low computational cost and model parameter count.

This performance improvement primarily stems from several factors. First, the multi-scale convolutions capture rich spatial receptive field features at different scales, which, combined with low-frequency components extracted through the low-pass filtering effect of global average pooling, effectively reinforce the model’s capability to represent small-sized, and morphologically diverse cable targets. In traditional convolutional fusion, global structural information tends to be progressively weakened due to the accumulation of high-frequency noise; however, the MC-GAP module leverages GAP’s spatial smoothing property to suppress high-frequency noise components while preserving low-frequency structural features, thereby aiding preservation of the global structure and crucial textures of targets, and improving robustness for recognizing subtle objects amid complex backgrounds.

Second, the introduction of the hybrid gating mechanism addresses the dynamic weighting balance between spatial domain features and low-frequency enhanced features (obtained through low-pass filtering). This mechanism combines global balance control (via learnable scalars α and β) with spatial adaptivity (via the Gate map), allowing the model to adjust contribution ratios at both coarse-grained and fine-grained levels according to scene and target characteristics. Specifically, the global parameters provide overall pathway balance, while the spatial-adaptive gate enables pixel-wise emphasis of frequency features in structurally important regions and spatial features in texture-rich areas. This dual-level adaptive mechanism not only reduces semantic conflicts and feature misalignment during feature fusion but also enhances the model’s ability to discriminate between similar slender targets. The residual connection further prevents feature degradation and preserves the integrity of the original image information.

We also observed that OPGW-DETR achieves high detection accuracy while ensuring low computational resource consumption and real-time inference, meeting the stringent requirements of low power consumption, efficiency, and precision in power line inspection systems.

Despite its strong performance, OPGW-DETR exhibits three key limitations. First, texture similarity between heart-shaped rings and nearby metallic hardware (e.g., clamps or bolts) can cause false positives or missed detections. Second, extremely small targets (<10–15 pixels) often lack sufficient discriminative features, leading to false negatives. Third, degraded image quality from motion blur, glare, or poor lighting further reduces detection reliability. These issues suggest promising avenues for future work, such as multi-frame fusion, illumination-robust training, and geometric priors.

Furthermore, despite the general expectation that larger models yield higher accuracy, we observe a performance plateau and even decline in OPGW-DETR-M and OPGW-DETR-X. This counterintuitive behavior stems from the confluence of three factors inherent to small-scale, high-stakes detection tasks. First, overconfidence under strict evaluation metrics: The mAP50–95 metric heavily penalizes localization errors at high IoU thresholds (≥0.75). In large-capacity models, classification confidence becomes decoupled from actual spatial precision, resulting in overconfident yet inaccurate predictions. Empirically, OPGW-DETR-X generates significantly more false positives at IoU ≥ 0.8 than its smaller counterparts, consistent with known miscalibration patterns in over-parameterized detectors [42,43]. Second, a severe mismatch between model capacity and dataset scale. OPGW-DETR-X contains 75.6 million parameters but is trained on only 3137 images, violating empirical scaling guidelines that recommend 10–20 samples per parameter for robust generalization [44]. This deficit leads to memorization of specific bounding box configurations rather than learning transferable spatial priors, manifesting as poor validation performance despite low training loss. Third, degradation of attention mechanisms at scale. Our architecture relies on Transformer-based modules (MC-GAP and hybrid gating) to enhance feature discrimination. However, in deeper variants, attention maps become diffuse, failing to localize subtle targets amid cluttered backgrounds. This phenomenon where increased capacity dilutes rather than sharpens focus is exacerbated by limited data diversity and aligns with recent observations in foundation model calibration under distribution shift [45]. Collectively, these effects explain why OPGW-DETR-S and -M achieve optimal performance: they provide sufficient capacity to leverage our architectural innovations without crossing into the overfitting and miscalibration regime. This insight underscores a critical design principle for UAV inspection systems, and moderate complexity often outperforms maximal scale when data is constrained.

In addition, this study emphasizes algorithmic innovation and achieves strong performance on standard GPU benchmarks, we recognize that practical UAV deployment necessitates empirical validation on embedded hardware. In future work, we plan to port our model to representative edge platforms such as the NVIDIA Jetson Xavier NX or AGX Orin, leveraging TensorRT with INT8 quantization to measure real-world metrics, including end-to-end inference latency (ms), frames per second (FPS), and power consumption (W), under realistic flight conditions. Our lightweight architecture and reduced reliance on high-frequency noise make it particularly well-suited for such resource-constrained environments. We view this algorithmic foundation as a necessary precursor to hardware-aware co-design, which we will pursue in subsequent research.

## 6. Conclusions

This paper presented OPGW-DETR, a real-time, high-precision detector for OPGW optical cables and conventional ground wires in UAV-based power transmission line inspections, built on an improved D-FINE framework. By integrating a MC-GAP module and a hybrid gating mechanism, the model dynamically fuses spatial and low-frequency features, substantially improving the representation of elongated, twisted, and small cable targets. Experimental results show that our method surpasses existing approaches with comparable cost under low power and computational budgets, meeting real-time deployment requirements for power line inspection. Future work will focus on enhancing robustness to noise and occlusion in complex environments and exploring the integration of multimodal sensor data and more advanced contextual modeling techniques.

## Figures and Tables

**Table 1:** Dataset category distribution statistics. O1/O2 denote OPGW cable types; D1/D2 denote conventional ground wire types.

Category ID	Name	Training Set	Validation Set	Test Set	Total Instances
0	O1	1323 (34.9%)	448 (35.4%)	451 (35.0%)	2222
1	D1	1251 (33.0%)	413 (32.6%)	423 (32.8%)	2087
3	O2	681 (18.0%)	226 (17.8%)	236 (18.3%)	1143
4	D2	533 (14.1%)	180 (14.2%)	179 (13.9%)	892
Total	—	3788	1267	1289	6344

**Table 2:** Bounding box size distribution statistics (in pixels).

Statistic	Width (px)	Height (px)	Area (px^2^)	Aspect Ratio
Minimum	2.1	1.8	7.4	0.08
5th Percentile	12.4	3.1	48.2	1.87
25th Percentile	18.3	4.2	89.3	2.47
Median (50%)	45.7	7.8	428.6	5.86
75th Percentile	98.4	15.2	1847.3	12.31
95th Percentile	234.6	38.9	8562.1	28.45
Maximum	578.2	162.4	52,387.9	89.73
Mean	72.8	12.6	1986.4	9.87
Std. Dev.	84.3	14.9	4123.7	11.24
CV (%)	115.8%	118.3%	207.6%	113.9%

**Table 3:** Relative target size distribution (as percentage of total image area).

Relative Size	Area Range	Instance Count	Percentage	Description
Tiny Targets	<0.1%	1582	24.9%	Near resolution limit
Small Targets	0.1%–0.5%	3124	49.2%	Primary detection objects
Medium Targets	0.5%–2.0%	1398	22.0%	Relatively easy to detect
Large Targets	>2.0%	240	3.8%	Close-range captures

**Table 4:** Spatial distribution of bounding box centers (normalized coordinates in [0,1]).

Image Region	X-Range	Y-Range	Count	Percentage
Center Region	0.33–0.67	0.33–0.67	2847	44.9%
Upper Center	0.33–0.67	0.00–0.33	523	8.2%
Lower Center	0.33–0.67	0.67–1.00	687	10.8%
Left Center	0.00–0.33	0.33–0.67	412	6.5%
Right Center	0.67–1.00	0.33–0.67	398	6.3%
Corner Regions	—	—	1477	23.3%

**Table 5:** Distribution of bounding boxes per image.

Boxes per Image	Image Count	Percentage	Scene Characteristics
1 box	1287	41.0%	Single conductor/long distance/occlusion
2 boxes	1024	32.6%	Typical dual-wire configuration
3 boxes	537	17.1%	Multi-span segments/complex towers
4 boxes	198	6.3%	Tower connection points/multi-circuit
≥5 boxes	91	2.9%	Extremely complex scenarios

**Table 6:** Detailed Configuration Parameters for Different Model Sizes.

Model Scale	Backbone Size	Returned Stages	$\left(C_{1},C_{2},C_{3}\right)$	$\left(s_{1},s_{2},s_{3}\right)$	*D*
N	B0	$S_{3}$, $S_{4}$	(512, 1024, –)	(16, 32, –)	128
S	B0	$S_{2}$, $S_{3}$, $S_{4}$	(256, 512, 1024)	(8, 16, 32)	256
M	B2	$S_{2}$, $S_{3}$, $S_{4}$	(384, 768, 1536)	(8, 16, 32)	256
X	B5	$S_{2}$, $S_{3}$, $S_{4}$	(512, 1024, 2048)	(8, 16, 32)	384

**Table 7:** Comprehensive comparison of hardware-level energy efficiency.

Model	GPU Power (W)	Avg. Memory (MB)	GPU Utilization (%)
	Peak/Idle		Peak/Idle
RT-DETR-R50	280/153	15,130	98/53
RT-DETR-R101	280/158	18,252	100/55
RT-DETR-X	282/169	18,138	100/57
UAV-DETR-Ev2	278/168	10,544	100/42
UAV-DETR-R18	246/151	9044	100/39
UAV-DETR-R50	259/159	15,526	100/43
YOLOv9-T	175/110	3006	47/22
YOLOv9-S	225/116	3688	62/39
YOLOv10-S	231/118	4422	77/50
YOLOv10-N	200/115	3510	57/42
D-FINE-N	233/68	6408	72/23
D-FINE-S	246/66	10,524	79/17
OPGW-DETR-N (Ours)	265/66	7584	78/27
OPGW-DETR-S (Ours)	268/66	18,376	99/32

**Table 8:** Performance Comparison of Various DETR and YOLO Models on OPGW Dataset.

Model	InputSize	mAP_50_	mAP_50∼95_	Param(M)	GFLOPs
RT-DETR-R50 [28]	640 × 640	70.9	45.2	40.8	130.5
RT-DETR-R101 [28]	640 × 640	72.8	46.7	58.9	191.4
RT-DETR-X [28]	640 × 640	74.1	46.3	65.5	222.5
UAV-DETR-Ev2 [30]	640 × 640	71.2	43.0	12.6	44.0
UAV-DETR-R18 [30]	640 × 640	72.6	44.7	20.5	73.9
UAV-DETR-R50 [30]	640 × 640	75.4	47.1	43.4	166.4
YOLOv9-T [40]	640 × 640	68.5	41.7	1.9	7.9
YOLOv9-S [40]	640 × 640	72.8	46.8	6.9	27.4
YOLOv10-S [41]	640 × 640	69.6	44.3	7.7	24.8
YOLOv10-N [41]	640 × 640	64.0	39.0	2.6	8.4
OPGW-DETR-N (Ours)	640 × 640	71.3	42.2	4.8	9.7
OPGW-DETR-S (Ours)	640 × 640	78.5	50.6	17.2	68.9
OPGW-DETR-M (Ours)	640 × 640	78.3	49.8	25.8	100.4
OPGW-DETR-X (Ours)	640 × 640	77.3	48.8	75.6	301.3

**Table 9:** Results of The Ablation Study ("✓" indicates the module is selected).

Baseline	MC-GAP	Gate	Params(M)	GFLOPs	mAP_50_	mAP_50∼95_
✓			9.7	24.8	74.6	46.3
✓	✓		17.1	67.8	78.4	49.4
✓	✓	✓	17.2	68.9	78.5	50.6

**Table 10:** Optimal Hybrid Gating Parameter Configurations.

Features	Level	$\alpha^{*}$	$\beta^{*}$	$\alpha^{*}$/$\beta^{*}$
$F_{S_{2}}$	Low	0.58	1.33	0.43
$F_{S_{3}}$	Mid	0.17	1.33	0.13
$F_{S_{4}}$	High	0.05	0.73	0.07

**Table 11:** Comprehensive comparison of frequency-selective metrics.

Metric	Projected	UAV-Enhanced	Δ Relative
Low-freq Energy (%)	93.0%	97.4%	+4.7%
90% Energy Cutoff (${\mathrm{px}}^{-1}$)	6	1	–83.3%
High-freq Energy (%)	2.2%	0.8%	–63.6%
Spectral Centroid (${\mathrm{px}}^{-1}$)	2.33	0.88	–62.3%
Spectral Spread (${\mathrm{px}}^{-1}$)	6.82	4.28	–37.3%
Mid-freq Energy (%)	4.8%	1.8%	–62.8%

## Data Availability

The data presented in this study are available on request from the corresponding author due to the data being part of an ongoing study.
